# Identifying Unique Subgroups of High-Cost Patients With Schizophrenia: A Population-Based Study Using Latent Class Analysis

**DOI:** 10.1177/11786329231183317

**Published:** 2023-06-24

**Authors:** Andrew J Stewart, Scott B Patten, Kirsten M Fiest, Tyler S Williamson, James P Wick, Paul E Ronksley

**Affiliations:** 1 Department of Community Health Sciences, Cumming School of Medicine, University of Calgary, AB, Calgary, Canada; 2 Department of Medicine, Cumming School of Medicine, University of Calgary, Calgary, AB, Canada

**Keywords:** Schizophrenia, comorbidity, health services research, economic analysis

## Abstract

Schizophrenia does not present uniformly among patients and as a result this patient population is characterized by a diversity in the type and amount of healthcare supports needed for daily functioning. Despite this, little work has been completed to understand the heterogeneity that exists among these patients. In this work we used a data-driven approach to identify subgroups of high-cost patients with schizophrenia to identify potentially actionable interventions for the improvement of outcomes and to inform conversations on how to most efficiently allocate resources in an already strained system. Administrative health data was used to conduct a retrospective analysis of “high-cost” adult patients with schizophrenia residing in Alberta, Canada in 2017. Costs were derived from inpatient encounters, outpatient primary care and specialist encounters, emergency department encounters, and drug costs. Latent class analysis was used to group patients based on their unique clinical profiles. Latent class analysis of 1659 patients revealed the following patient groups: (1) young, high-needs males early in their disease course; (2) actively managed middle-aged patients; (3) elderly patients with multiple chronic conditions and polypharmacy; (4) unstably housed males with low treatment rates; (5) unstably housed females with high acute care use and low treatment rates. This taxonomy may be used to inform policy, including the identification of interventions most likely to improve care and reduce health spending for each subgroup.

## Background

A small proportion of patients account for a disproportionate amount of health spending in countries with advanced healthcare systems. In North America, patients in the top 1% of healthcare spending account for approximately 25-40% of total healthcare expenditures.^1,2^ Prior studies have described the clinical and demographic profiles of these “high-cost” patients and have found an over-representation of mental health diagnoses.^3-5^ Unfortunately, the heterogeneous nature of this patient population makes it difficult to develop and implement strategies to improve care and curb health spending for all high-needs, high-cost patients.^6^ This is especially true for patients with schizophrenia as this disorder is well known for its association with high levels of disability and healthcare utilization.^7-9^ Further, the economic costs of providing mental health services varies widely between patients.^10^

There is growing interest in stratifying high-cost patients into subgroups to facilitate the application of targeted inventions.^11-15^ Common approaches used to characterize high-cost patient subgroups have focused on identification of independent demographic and clinical factors through regression modeling.^3,16-18^ A limitation of this approach is that it assumes these variables are discrete representations of risk factors and may not account for the fact that interrelated combinations of these variables may better represent the underlying mechanisms that drive patient need. Patient health data can be analyzed from this perspective using latent class analysis (LCA) which is a statistical method for identifying unmeasured class membership among subjects.^19^ This technique allows researchers to identify and accurately enumerate the number of high-cost patient groups (or classes), estimate the prevalence of the groups, and highlight the characteristics that define class membership.^20^

Given the patient and health system burden associated with schizophrenia, there is a need to better match unique patient needs with available interventions to improve quality of life, patient outcomes, and address growing healthcare spending. We used LCA to develop a taxonomy of unique subgroups of high-cost patients with schizophrenia living in Alberta, Canada which may be targetable by specific interventions. Our goal was to use a data-driven approach to better understand the heterogeneity among high-cost patients with schizophrenia (ie, those in the upper 95th percentile of annual healthcare spending). Within the research community there exists a long-term ambition of identifying interventions most likely to improve care and reduce health spending for different subgroups of high-cost patients with schizophrenia. As such, we also matched potential interventions to each group identified within our LCA to not only demonstrate the utility of this proposed taxonomy, but to drive the research agenda forward in this important area.

## Methods

### Data

This study used administrative health data from a 10-year patient cohort (January 1, 2008 and December 31, 2017) of high-cost adult patients with schizophrenia residing in Alberta, Canada. A detailed description of the cohort creation has been published previously.^21,22^ Briefly, a case ascertainment algorithm was used to identify patients who received care for schizophrenia between 2008 and 2017. The definition for schizophrenia used was: “1 hospitalization or 2 physician billing claims in 2 years or less associated with an F20.X, F21.X, F23.2 or F25.X ICD-10 code or a ‘295.X’ ICD-9 code.”^23-25^ This algorithm has a reported positive predictive value of 87% and a sensitivity of 87%.^23^ Patients with schizophrenia entered the cohort on the date of their first schizophrenia-specific ICD code and were followed until death, outmigration, or end of study follow-up (December 31, 2017). Finally, a “high-cost” patient cohort was created as a subset of the total patient population for the period of January 1 to December 31, 2017 (1 year cohort) to remove secular trends in patient demographics and health spending observed in prior work by our team.^21,22^ Given the exploratory nature of this investigation, to facilitate comparison with existing high-cost literature,^1,5,26,27^ a “high-cost” patient with schizophrenia was defined as an individual with annual direct healthcare costs in the 95th percentile or greater.

#### Independent variables

Risk factors for high-cost status considered in this work were informed by the Gelberg-Andersen Behavioral Model for Vulnerable Populations.^28^ This model takes a holistic approach in describing factors associated with healthcare utilization and includes 3 domains with special attention to exposures particularly relevant to vulnerable populations: Environmental Factors (eg, geography, social environment), Population Characteristics (eg, pre-disposing, enabling, and need factors), and Health Behaviors (eg, seeking specialist care, and other patterns of healthcare access). Demographic and clinical characteristics of all patients were extracted from the same administrative datasets. This included age, sex, and postal code (to derive estimates of population density). A proxy measure for housing stability was also generated by searching healthcare records for a record of homelessness (Z59 ICD-10 code), or a shelter-associated postal code. To obtain further detail on patient need, comorbidity profiles were determined through the use of 29 case-ascertainment algorithms defined by Tonelli et al.^23^ The proportion of patients with each unique chronic condition was determined and multimorbidity was defined and categorized based on the co-occurrence of these conditions (0, 1-2, 3+).

Information related to healthcare resource use was derived from the administrative data sources described above. This included number of hospitalizations, ED visits, and specialist visits in the prior year (categorized as 0, 1-2, 3+ encounters). Finally, annual prescription information (Anatomical Therapeutic Chemical (ATC) and date of prescription) was used to determine the number of unique pharmaceuticals prescribed to each patient within the calendar year. Individuals were exposed to “polypharmacy” if they were prescribed 5 to 9 unique medications and exposed to “extreme polypharmacy” if prescribed 10 or more medications.

#### Analytic Approach

LCA is a statistical method in which observed variables are used to categorize individuals into otherwise unobserved “classes” through patterns of conditional probabilities for each individual. As there is no gold standard for determining the number of latent classes that best fit a dataset, we followed model specification procedures used previously in the literature.^29^ This began with the specification of a model with 2 classes and adding classes iteratively until the Bayesian Information Criterion (BIC) were minimized while ensuring that models converged.^29^ The prevalence of demographic characteristics, measures of system use, specialty involvement, and various comorbidities, as well as the risk ratio of the characteristic’s prevalence in the group compared to the overall prevalence across all subgroups were tabulated to aid in the identification of the defining characteristics of each subgroup. The proportions of cost attributable to each type of encounter (inpatient, ED, outpatient, medication) as well as the costliest and most frequent diagnostic codes (hospitalizations, ED encounters, physician claims) and ATC codes (prescribed medications) within each subgroup were also determined using costing techniques described previously.^21,22^

All analyses were completed using STATA 16.^30^ This study follows the REporting of studies Conducted using Observational Routinely-collected Data (RECORD) statement^31^ (Supplemental A).

## Results

### High-cost population: Demographic characteristics and health system use

The 95th percentile for cost included 1659 “high-cost” Alberta patients over the age of 18 with schizophrenia in 2017. More males than females were present in the cohort (n = 931 vs 728). Middle-aged patients (40-69 years of age) comprised 47.8% of this cohort and geographically most patients were found to live in Alberta’s larger urban centers. High-cost patients were found to have high levels of material deprivation (25% in the most deprived quartile) and 70.5% had 3 or more chronic conditions (Table 1). The most common chronic conditions were depression, alcohol misuse, and hypertension (Supplemental B).

**Table 1.** Clinical and demographic characteristics of each latent class.

	Class 1 (n = 514)	Class 2 (n = 353)	Class 3 (n = 374)	Class 4 (n = 241)	Class 5 (n = 177)	Total Cohort (N = 1659)
Age						
18-29	323 (62.8%)	12 (3.4%)	0 (0.0%)	7 (2.9%)	23 (13.0%)	365 (22.0%)
30-39	132 (25.7%)	99 (28.0%)	0 (0.0%)	32 (13.3%)	43 (24.3%)	306 (18.4%)
40-69	59 (11.5%)	239 (67.7%)	191 (51.1%)	202 (83.8%)	111 (62.7%)	793 (47.8%)
70+	0 (0.0%)	12 (3.4%)	183 (48.9%)	0 (0.0%)	0 (0.0%)	195 (11.8%)
Sex						
Male	357 (69.5%)	191 (54.1%)	143 (38.2%)	240 (99.6%)	0 (0.0%)	931 (56.1%)
Female	157 (30.5%)	162 (45.9%)	231 (61.8%)	1 (0.4%)	177 (100%)	728 (43.9%)
Location of Residence						
Rural	54 (10.5%)	36 (10.2%)	36 (9.6%)	32 (13.3%)	21 (11.9%)	179 (10.8%)
Small	44 (8.6%)	29 (8.2%)	40 (10.7%)	19 (7.9%)	19 (10.7%)	151 (9.1%)
Medium	49 (9.5%)	30 (8.5%)	34 (9.1%)	22 (9.1%)	24 (13.6%)	159 (9.6%)
Urban	269 (52.3%)	222 (62.9%)	227 (60.7%)	149 (61.8%)	105 (59.3%)	972 (58.6%)
No Information	9 (1.8%)	6 (1.7%)	2 (0.5%)	4 (1.7%)	2 (1.1%)	23 (1.4%)
Unstably Housed	146 (28.4%)	0 (0.0%)	13 (3.5%)	100 (41.5%)	60 (33.9%)	319 (19.2%)
Annual ED visits						
0	80 (15.6%)	80 (22.7%)	36 (9.6%)	12 (5.0%)	9 (5.1%)	217 (13.1%)
1-2	152 (29.6%)	179 (50.7%)	140 (37.4%)	53 (22%)	25 (14.1%)	549 (33.1%)
3 or more	282 (54.9%)	94 (26.6%)	198 (52.9%)	176 (73%)	143 (80.8%)	893 (53.8%)
Annual Hospitalizations						
0	59 (11.5%)	80 (22.7%)	20 (5.3%)	5 (2.1%)	3 (1.7%)	167 (10.1%)
1-2	228 (44.4%)	270 (76.5%)	209 (55.9%)	59 (24.5%)	31 (17.5%)	797 (48.0%)
3 or more	227 (44.2%)	3 (0.8%)	145 (38.8%)	177 (73.4%)	143 (80.8%)	695 (41.9%)
Specialist Involvement in Care						
0	40 (7.8%)	29 (8.2%)	5 (1.3%)	1 (0.4%)	3 (1.7%)	78 (4.7%)
1-2	231 (44.9%)	119 (33.7%)	62 (16.6%)	36 (14.9%)	34 (19.2%)	482 (29.1%)
3 or more	243 (47.3%)	205 (58.1%)	307 (82.1%)	204 (84.6%)	140 (79.1%)	1099 (66.2%)
Material Deprivation						
Missing Address	23 (4.5%)	32 (9.1%)	58 (15.5%)	40 (16.6%)	20 (11.3%)	173 (10.4%)
First Quintile	80 (15.6%)	49 (13.9%)	42 (11.2%)	33 (13.7%)	21 (11.9%)	225 (13.6%)
Second Quintile	57 (11.1%)	40 (11.3%)	23 (6.1%)	11 (4.6%)	18 (10.2%)	149 (9.0%)
Third Quintile	77 (15%)	55 (15.6%)	63 (16.8%)	24 (10.0%)	27 (15.3%)	246 (14.8%)
Fourth Quintile	72 (14%)	58 (16.4%)	65 (17.4%)	49 (20.3%)	32 (18.1%)	276 (16.6%)
Fifth Quintile	116 (22.6%)	89 (25.2%)	88 (23.5%)	69 (28.6%)	53 (29.9%)	415 (25%)
Antipsychotic Treatment						
None	154 (30%)	132 (37.4%)	235 (62.8%)	105 (43.6%)	79 (44.6%)	705 (42.5%)
Oral	334 (65%)	202 (57.2%)	135 (36.1%)	127 (52.7%)	94 (53.1%)	892 (53.8%)
Injection	26 (5.1%)	19 (5.4%)	4 (1.1%)	9 (3.7%)	4 (2.3%)	62 (3.7%)
Oral + Injectable	17 (3.3%)	11 (3.1%)	3 (0.8%)	7 (2.9%)	2 (1.1%)	40 (2.4%)
Polypharmacy						
0 Unique ATC’s	0 (0.0%)	0 (0.0%)	0 (0.0%)	0 (0.0%)	0 (0.0%)	0 (0.0%)
1-4 Unique ATC’s	195 (37.9%)	88 (24.9%)	39 (10.4%)	19 (7.9%)	13 (7.3%)	354 (21.3%)
5-9 Unique ATC’s (Polypharmacy)	171 (33.3%)	87 (24.6%)	46 (12.3%)	42 (17.4%)	17 (9.6%)	363 (21.9%)
10 or more Unique ATC’s (Extreme Polypharmacy)	148 (28.8%)	178 (50.4%)	289 (77.3%)	180 (74.7%)	147 (83.1%)	942 (56.8%)
Comorbidity						
0 Comorbidities	26 (5.1%)	8 (2.3%)	0 (0.0%)	0 (0.0%)	0 (0.0%)	34 (2.0%)
1-2 Comorbidities	327 (63.6%)	108 (30.6%)	2 (0.5%)	12 (5.0%)	6 (3.4%)	455 (27.4%)
3 or more Comorbidities	161 (31.3%)	237 (67.1%)	372 (99.5%)	229 (95.0%)	171 (96.6%)	1170 (70.5%)

The frequency of ED visits in this population was high with 54% of the high-cost cohort utilizing emergency services 3 or more times in the prior year. In contrast, hospitalizations were less frequent with 58% of patients being hospitalized 2 or fewer times over the same period. Polypharmacy was found to be extremely high with 1305 (78.7%) patients being prescribed 5 or more unique drugs. Over half of the high-cost cohort (58.5%) were prescribed antipsychotic treatments which were most commonly oral formulations (Table 1). Treatment of schizophrenia/schizoaffective disorders ranked highly within the top 10 most frequently used administrative codes associated with each latent class (Supplemental C).

### Derivation of high-cost subgroups through latent class analysis

LCA identified 5 unique classes of patients. High-cost patients were well-represented across each of the latent classes with the largest class including 514 (31.0%) patients and the smallest class including 177 (10.7%) patients. These 5 subgroups predominantly included individuals that were:

(1)  young, high-needs males early in their disease course,
(2)   actively managed middle-aged patients,
(3)  elderly patients with multiple chronic conditions and extreme polypharmacy,
(4)  unstably housed males with low treatment rates,
(5)   unstably housed females with high acute care use and low treatment rates.

**Figure 1.**
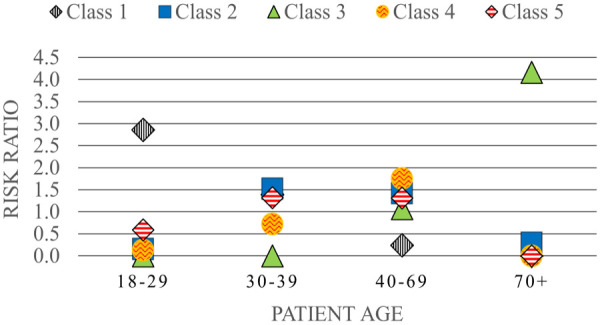
Age distribution of patients stratified by latent class (risk ratios)*. *Risk ratio of the prevalence of each age stratum in each class compared to the overall prevalence across all subgroups (ie, the entire high-cost cohort).

**Figure 2.**
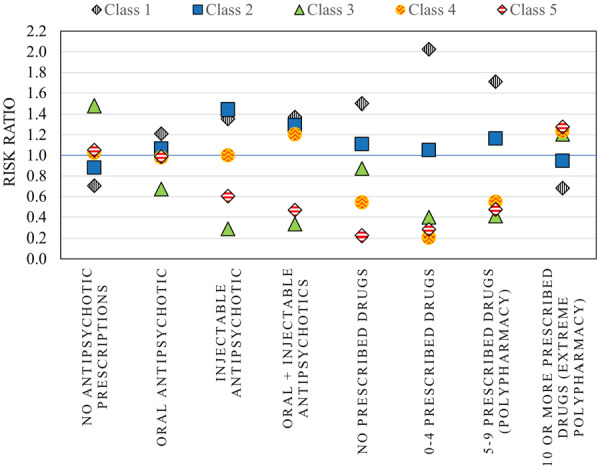
Prescription patterns stratified by latent class (risk ratios)*. *Risk ratio of the prevalence of each treatment type in each class compared to the overall prevalence across all subgroups (ie, the entire high-cost cohort).

**Figure 3.**
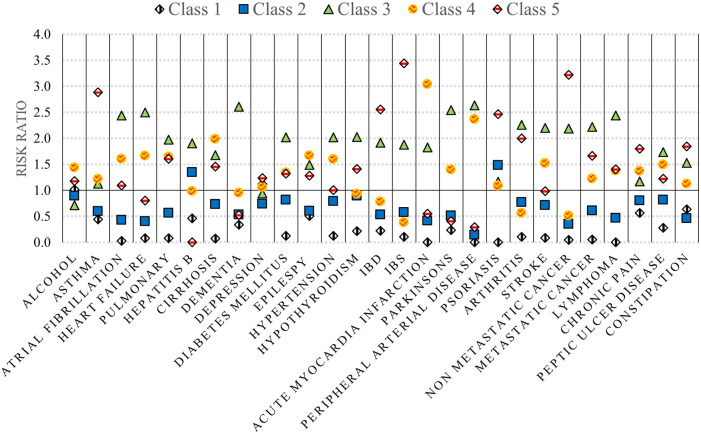
Comorbidity prevalence stratified by latent class (risk ratios)*. *Risk ratio of the prevalence of each comorbidity in each class compared to the overall prevalence across all subgroups (ie, the entire high-cost cohort).

**Figure 4.**
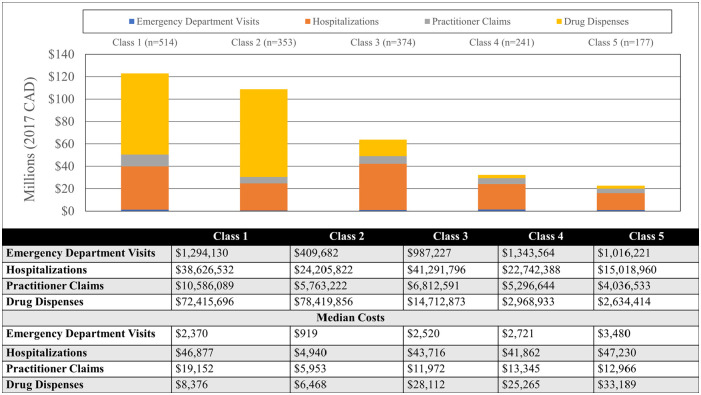
Direct healthcare costs stratified by latent class and spending category.

The unique demographic and clinical profiles for each of these classes are summarized in Table 1 and Figures 1 to 3 and stratified costs for each class are summarized in Figure 4. We provide a summary of the unique characteristics that define each latent class below.

### Class 1: Young, high-needs patients early in their disease course

Class 1 was the most prevalent class identified. Younger high-cost patients were best represented in this subgroup with 88.5% under the age of 30. This class was also heavily skewed toward males (69.5%). Approximately one quarter were unstably housed and 36.9% had no ED encounters or hospitalizations (35.3%) in the prior year. Over two-thirds (68%) were exposed to polypharmacy and included the highest numbers of patients prescribed oral and injectable antipsychotics.

### Class 2: Actively managed middle-aged patients

Class 2 had the greatest representation of middle-aged patients (aged 40-69) and included an almost equal proportion of males and females. Healthcare utilization was the lowest of the classes identified. Further, they had the lowest risk of being unstably housed, and the lowest risk for high acute care use and specialist involvement in their care relative to other high-cost classes. This was corroborated by low risk ratios for most comorbidities with only Class 1 having lower risk estimates (Figure 3). This class also had a high proportion receiving injectable antipsychotic medications.

### Class 3: Elderly patients with multiple chronic conditions and polypharmacy

This class represented the second highest number of high-cost patients (n = 374). The age and sex profiles captured within this class were distinct from those in Classes 1 and 2. Most (93.8%) were aged 70 years or older and did not include any patients under the age of 40 (Figure 1). There was a higher proportion of females in this class and 95.7% were stably housed. The risk ratio of most chronic conditions was higher than all other classes particularly for dementia and cardiovascular comorbidities. In addition, a high level of “extreme polypharmacy” (10 or more unique drug prescriptions) was observed (Figure 2). The top 10 most frequent diagnostic codes for ED visits, hospitalizations, and physician claims reflected a different profile than those observed for other classes where these patients received more care for comorbidity and age-related pathologies (eg, rehabilitative care, urinary tract infections, and chest pain) (Supplemental C).

### Class 4: Unstably housed males with low treatment rates

Class 4 was entirely composed of males apart from the inclusion of one female patient. They were more likely to be older (aged 40-69) and had the highest risk of being homeless/unstably housed (Table 1). They also had lower risk of receiving medications for their underlying chronic conditions (Figure 2) and a higher risk of being recurrent users of acute healthcare services (ED visits and hospitalizations) relative to other high-cost classes. Patients within this class were more commonly treated for alcohol toxicity and other stimulants compared with the other classes (Supplemental C).

### Class 5: Unstably housed females with high acute care use and low treatment rates

While Classes 4 and 5 appeared most similar in profile, there were unique characteristics that distinguished this subgroup. This was the smallest group of high-cost patients (n = 177) and composed entirely of female patients. The risk of being homeless/unstably housed was slightly lower than Class 4 but use of ED services and hospitalizations was highest. Comorbidity profiles also differed with the highest risk for gastrointestinal conditions such as irritable bowel syndrome and disease (IBS and IBD) in addition to non-metastatic cancer, asthma, and chronic pain. They also had the lowest risk of receiving any prescribed medications for their chronic conditions and the second lowest risk of receiving injectable antipsychotics compared with the other classes.

### Cost distribution

Figure 4 displays direct healthcare costs stratified by spending category and latent class membership. Costs were heavily skewed and median costs ranged from $90121.06 (Q1-Q3: $74125.63−$159169.80) in Class 2 (actively managed middle-aged patients) to $109336.40 (Q1-Q3: $80537.89−$180157.50) in Class 3 (elderly patients with multiple chronic conditions and polypharmacy). Class 1 was associated with the highest estimated cumulative spending with Classes 4 and 5 representing the lowest cumulative spending. The greatest contributors to cost for Classes 1 and 2 were prescribed medications and the greatest contributors to costs for the remaining classes were hospitalizations.

## Discussion

In this population-based study, we used administrative healthcare data and LCA to develop a taxonomy of 5 unique subgroups of patients with schizophrenia which may be targetable by specific interventions. These 5 classes have distinct demographic and clinical characteristics, patterns of healthcare use, and health behaviors. Further, this data-driven approach provides key insights into the different profiles of high-cost patients with schizophrenia and can inform care strategies based on their disease course, healthcare needs, and social circumstances.

While little work has focused on high-cost mental health patients,^4^ many of our findings are supported by previous literature. Rosella et al found that being a high-cost patient was strongly associated with being older and having multiple chronic conditions^16^ and Wang et al found that comorbidity was an important predictor of high-cost status in this patient population.^32^ While studies such as these have identified independent risk factors for high-cost status, latent class methodologies allow us to develop a taxonomy that describe how different predictors co-exist to create unique patient subgroups. This permits a more precise approach to improving care for this population by offering avenues to prioritize more individualized treatment strategies. In the following paragraphs we provide our interpretation of these profiles along with a discussion of interventions/supports that offer promise in shifting these patients away from high use of healthcare resources while potentially addressing quality of life and overall health outcomes.

### Class 1: Young, high-needs males early in their disease course

Patients in this group were characterized as being younger, living with less comorbidity, but having highly diverse healthcare needs (as characterized by high reliance on inpatient and outpatient services). Pharmaceutical treatment costs were also found to be the highest spending category in this class. From a policy perspective, generic formulations may offer significant cost savings while still delivering the same benefits of the currently available patented drugs. Unfortunately, generic formulations aren’t likely to become available for several years and availability of competing formulations is scarce in Canada.^33^ Fortunately, there is some political will for the Implementation of National Pharmacare and the Schizophrenia Society of Canada recommends the pursuit of this goal.^34^ Economic analyses also suggest that combining the purchasing power of multiple provinces under a national framework may help realize reductions in Canadian prescription drug expenditures.^35^

This group of high-cost patients is also well positioned to benefit from preventative medicine interventions. The Lancet Psychiatry Commission recommends that “among those with mental illness, a healthy lifestyle should ideally be adopted in the early stages of illness to build health habits and to protect physical health as much as possible.”^36^ Some transdiagnostic case-management supports currently exist in Alberta which may facilitate this. For example, patients with chronic psychotic disorders can access the Adult Psychosis program which connects them with a network of living skills instructors, occupational therapists, psychiatrists, psychologists, registered nurses, registered psychiatric nurses and social workers.^37^ There may be value in investigating options for expanding this program to additional sites, expanding the variety of professional supports available beyond those commonly associated with mental healthcare (eg, dieticians, physiotherapists), and opening new referral pathways for younger patients.

### Class 2: Actively managed middle-aged patients

Patients in class 2 were generally older and appeared to present a more uniform profile when it came to healthcare resource utilization. There appeared to be reduced reliance on acute healthcare services and an increased reliance on more expensive pharmaceutical treatments (ie, injectable antipsychotics). Thus, patients in this subgroup also stand to benefit from the recommendations made above. This includes both the use of case managers to facilitate/organize transdiagnostic approaches to treatment along with structural changes to the way pharmaceutical costs are negotiated in Canada.

### Class 3: Elderly patients with multiple chronic conditions and polypharmacy

Patients in this group were generally over the age of 70 with an increased reliance on acute healthcare services, increased levels of comorbidity, and polypharmacy. It is well-established that older patients with schizophrenia have lower levels of community integration than age-matched peers^38^ which may be a contributing factor to this trend. Programs that aim to improve community integration of patients are offered in Alberta, but their scope is limited. For example, the Schizophrenia Society of Alberta fosters peer connections though a province-wide senior’s phone peer support group that meets weekly. Older patients with schizophrenia may benefit from technology skills training that would allow them to access additional formal and semi-formal ways to connect and interact with their community and the healthcare system. In addition, patients with schizophrenia are faced with new challenges with the onset of age-related morbidity. The delivery of personalized medicine is time intensive and given pressures that already exist within the healthcare system these needs may be best met by dedicated case management and a focus on “upstream interventions” associated with preventative medicine.

### Classes 4 and 5: Unstably housed individuals with high acute care use and low treatment rates

Classes 4 and 5 were both characterized by high levels of homelessness/unstable housing and high acute care use while accessing prescribed antipsychotics at lower rates. However, classes 4 and 5 could be differentiated from one another primarily through sex with class 4 being >99% males and class 5 only including females. Given the reliance of these high-cost populations on acute care services we believe that these groups would benefit the most from Assertive Community Treatment (ACT). Prior meta-analyses have found that ACT is effective at reducing hospitalizations which in turn improves patient quality of life and addresses growing inpatient spending in this subset of patients.^39,40^ In fact, studies on ACT have found increased levels of treatment engagement and reductions in those with homelessness^41-43^ which would shift the profile of these patients closer to those seen in class 2. As class 2 remains a high-cost group this shift may not curb health spending, but from a patient-centric perspective this shift may remain a valuable outcome. In addition, our review of hospital service codes suggest that male patients may benefit from interventions focused on harm reduction for alcohol and stimulants.

The high proportion of unstably housed patients in these groups suggest that housing remains an issue. The Schizophrenia Society of Alberta provides some support in this area providing supportive housing to a small number of patients with schizophrenia at a cost of approximately $72 per day.^44^ Unfortunately, this is only a subset of Alberta’s homeless schizophrenia population, and our cohort suggests that patients continue to fall through the gaps. The benefits associated with the provision of housing supports for this population are well established,^45^ however associated costs cannot be ignored. For example, after speaking with industry experts, Phan estimated that High Supportive Housing is associated with a cost of $130 to 150 per day.^46^ There is a clear need for discussions on the value of increasing the capacity of programs such as those delivered by the Schizophrenia Society of Alberta. However, this would require rigorous evaluation to determine cost effectiveness and long-term effects on patient outcomes.

## Strengths & Limitations

A key strength of this study is its use of detailed and diverse administrative data sources allowing for identification of high-cost individuals at a provincial level. Further, the use of LCA allows for a more precise taxonomy than many other studies in this area, while ensuring that the resulting groups have face validity, are clinically meaningful, and offer opportunity for targeted interventions. Despite its strengths, the results of the study should be interpreted considering its limitations. Our taxonomy is driven by the data sources used as its inputs. As a result, important measures of the social determinants of health such as education level and food insecurity were not captured and may have missed key latent classes. Further, while the groupings are mutually exclusive, there are characteristics that overlap across all 5 classes and patients may shift between classes as their disease progresses, their social and medical circumstances change, or as they age. We also recognize that this latent class analysis was conducted on a group of patients classified as “high-cost” in a single year and patients can move between high-cost and non-high-cost states over time.^47^ Thus, further insights into the variability of LCA groupings in this patient population may be gleaned by contrasting our findings with LCA groupings generated from cohorts of patients classified as “high-cost” across multiple years (ie, persistently high-cost patients). This may improve the chances that targeted interventions would truly affect health spending and more importantly patient outcomes. However, identifying the key characteristics of each subgroup, even for a 1 year cohort, highlights areas for potential cost containment at a specific point in time and is highly relevant from both a clinical and public health perspective. Finally, our taxonomy hinges on our measure of “high-cost” status. While we did not conduct sensitivity analyses, future research may consider exploring alternative cost cut-offs (eg, upper 10% of upper 2.5% of patients) or different high-cost definitions that rely on frequencies of healthcare encounters (ie, number of hospitalizations or emergency department visits) to determine the validity of our 5 proposed subgroups of high-cost patients with schizophrenia.

## Conclusions

The results of this analysis indicate that high-cost patients with schizophrenia are not a homogenous group, and several distinct subgroups exist. Our data-driven approach using LCA was able to uncover 5 classes. When aligned with available interventions such as case-management, policy aimed at reducing costs of injectable antipsychotics, assertive community treatment, housing initiatives, and improved access to supports for elderly patient may help us realize cost savings in the vulnerable population while improving health outcomes.

## Supplemental Material

sj-docx-1-his-10.1177_11786329231183317 – Supplemental material for Identifying Unique Subgroups of High-Cost Patients With Schizophrenia: A Population-Based Study Using Latent Class Analysis Click here for additional data file. Supplemental material, sj-docx-1-his-10.1177_11786329231183317 for Identifying Unique Subgroups of High-Cost Patients With Schizophrenia: A Population-Based Study Using Latent Class Analysis by Andrew J Stewart, Scott B Patten, Kirsten M Fiest, Tyler S Williamson, James P Wick and Paul E Ronksley in Health Services Insights

sj-docx-2-his-10.1177_11786329231183317 – Supplemental material for Identifying Unique Subgroups of High-Cost Patients With Schizophrenia: A Population-Based Study Using Latent Class Analysis Click here for additional data file. Supplemental material, sj-docx-2-his-10.1177_11786329231183317 for Identifying Unique Subgroups of High-Cost Patients With Schizophrenia: A Population-Based Study Using Latent Class Analysis by Andrew J Stewart, Scott B Patten, Kirsten M Fiest, Tyler S Williamson, James P Wick and Paul E Ronksley in Health Services Insights

sj-docx-3-his-10.1177_11786329231183317 – Supplemental material for Identifying Unique Subgroups of High-Cost Patients With Schizophrenia: A Population-Based Study Using Latent Class Analysis Click here for additional data file. Supplemental material, sj-docx-3-his-10.1177_11786329231183317 for Identifying Unique Subgroups of High-Cost Patients With Schizophrenia: A Population-Based Study Using Latent Class Analysis by Andrew J Stewart, Scott B Patten, Kirsten M Fiest, Tyler S Williamson, James P Wick and Paul E Ronksley in Health Services Insights
