# Supplementary material for: Identifying Unique Subgroups of High-Cost Patients With Schizophrenia: A Population-Based Study Using Latent Class Analysis
Source: Health Serv Insights. 2023 Jun 24;16:11786329231183317. doi: 10.1177/11786329231183317 (PMC10291413; doi:10.1177/11786329231183317)
Supplement: sj-docx-2-his-10.1177_11786329231183317 – Supplemental material for Identifying Unique Subgroups of High-Cost Patients With Schizophrenia: A Population-Based Study Using Latent Class Analysis [file sj-docx-2-his-10.1177_11786329231183317.docx]

Supplement B: Intraclass Comorbidity Prevalence

|  | Class 1  (n=514) | Class 2  (n=353) | Class 3  (n=374) | Class 4  (n=241) | Class 5  (n=177) | Total  (N=1659) |
| --- | --- | --- | --- | --- | --- | --- |
| Alcohol Misuse | 238  (46.3%) | 143  (40.5%) | 121  (32.4%) | 157  (65.1%) | 95  (53.7%) | 754  (45.4%) |
| Asthma | 30  (5.8%) | 28  (7.9%) | 56  (15%) | 39  (16.2%) | 68  (38.4%) | 221  (13.3%) |
| Atrial Fibrillation | 1  (0.2%) | 11  (3.1%) | 66  (17.6%) | 28  (11.6%) | 14  (7.9%) | 120  (7.2%) |
| Heart Failure | 5  (1.0%) | 17  (4.8%) | 112  (29.9%) | 48  (19.9%) | 17  (9.6%) | 199  (12%) |
| Pulmonary | 12  (2.3%) | 57  (16.1%) | 211  (56.4%) | 113  (46.9%) | 81  (45.8%) | 474  (28.6%) |
| Hepatitis b | 1  (0.2%) | 2  (0.6%) | 3  (0.8%) | 1  (0.4%) | 0  (0.0%) | 7  (0.4%) |
| Cirrhosis | 1  (0.2%) | 7  (2.0%) | 17  (4.5%) | 13  (5.4%) | 7  (4.0%) | 45  (2.7%) |
| Dementia | 45  (8.8%) | 48  (13.6%) | 250  (66.8%) | 59  (24.5%) | 24  (13.6%) | 426  (25.7%) |
| Depression | 457  (88.9%) | 207  (58.6%) | 279  (74.6%) | 204  (84.6%) | 174  (98.3%) | 1321  (79.6%) |
| Diabetes Mellitus | 18  (3.5%) | 79  (22.4%) | 207  (55.3%) | 87  (36.1%) | 64  (36.2%) | 455  (27.4%) |
| Epilepsy | 40  (7.8%) | 33  (9.3%) | 86  (23%) | 62  (25.7%) | 35  (19.8%) | 256  (15.4%) |
| Hypertension | 27  (5.3%) | 118  (33.4%) | 319  (85.3%) | 163  (67.6%) | 75  (42.4%) | 702  (42.3%) |
| Hypothyroidism | 22  (4.3%) | 62  (17.6%) | 149  (39.8%) | 44  (18.3%) | 49  (27.7%) | 326  (19.7%) |
| Inflammatory Bowel Disease | 3  (0.6%) | 5  (1.4%) | 19  (5.1%) | 5  (2.1%) | 12  (6.8%) | 44  (2.7%) |
| Irritated Bowel Syndrome | 3  (0.6%) | 11  (3.1%) | 38  (10.2%) | 5  (2.1%) | 33  (18.6%) | 90  (5.4%) |
| Acute Myocardia Infarction | 0  (0.0%) | 3  (0.8%) | 14  (3.7%) | 15  (6.2%) | 2  (1.1%) | 34  (2.0%) |
| Parkinson’s | 10  (1.9%) | 15  (4.2%) | 79  (21.1%) | 28  (11.6%) | 6  (3.4%) | 138  (8.3%) |
| Peripheral Arterial Disease | 0  (0.0%) | 1  (0.3%) | 19  (5.1%) | 11  (4.6%) | 1  (0.6%) | 32  (1.9%) |
| Psoriasis | 0  (0.0%) | 12  (3.4%) | 10  (2.7%) | 6  (2.5%) | 10  (5.6%) | 38  (2.3%) |
| Arthritis | 2  (0.4%) | 10  (2.8%) | 31  (8.3%) | 5  (2.1%) | 13  (7.3%) | 61  (3.7%) |
| Stroke | 7  (1.4%) | 39  (11%) | 128  (34.2%) | 57  (23.7%) | 27  (15.3%) | 258  (15.6%) |
| Non-Metastatic Cancer | 1  (0.2%) | 5  (1.4%) | 33  (8.8%) | 5  (2.1%) | 23  (13%) | 67  (4.0%) |
| Metastatic Cancer | 1  (0.2%) | 8  (2.3%) | 31  (8.3%) | 11  (4.6%) | 11  (6.2%) | 62  (3.7%) |
| Lymphoma | 0  (0.0%) | 2  (0.6%) | 11  (2.9%) | 4  (1.7%) | 3  (1.7%) | 20  (1.2%) |
| Chronic Pain | 68  (13.2%) | 67  (19%) | 103  (27.5%) | 78  (32.4%) | 75  (42.4%) | 391  (23.6%) |
| Peptic Ulcer Disease | 2  (0.4%) | 4  (1.1%) | 9  (2.4%) | 5  (2.1%) | 3  (1.7%) | 23  (1.4%) |
| Constipation | 36  (7%) | 18  (5.1%) | 63  (16.8%) | 30  (12.4%) | 36  (20.3%) | 183  (11.0%) |
