# Supplementary material for: Identifying Unique Subgroups of High-Cost Patients With Schizophrenia: A Population-Based Study Using Latent Class Analysis
Source: Health Serv Insights. 2023 Jun 24;16:11786329231183317. doi: 10.1177/11786329231183317 (PMC10291413; doi:10.1177/11786329231183317)
Supplement: sj-docx-3-his-10.1177_11786329231183317 – Supplemental material for Identifying Unique Subgroups of High-Cost Patients With Schizophrenia: A Population-Based Study Using Latent Class Analysis [file sj-docx-3-his-10.1177_11786329231183317.docx]

Supplement C: Prevalence of ICD Codes in Each Latent Class

***Latent Class 1: Young, High-needs Males Early in Their Disease Course***

| Rank | Code |  | Description | | Freq. | Percent |
| --- | --- | --- | --- | --- | --- | --- |
|  |  |  |  | |  |  |
| Hospitalizations | | | | | | |
| 1 | F209 |  | Schizophrenia, unspecified | | 335 | 25.02 |
| 2 | F29 |  | Unspecified nonorganic psychosis | | 109 | 8.14 |
| 3 | F259 |  | Schizoaffective disorder, unspecified | | 92 | 6.87 |
| 4 | F252 |  | Schizoaffective disorder, mixed type | | 67 | 5 |
| 5 | F155 |  | Mental and behavioural disorders due to use of other stimulants, including caffeine: Psychotic disorder | | 61 | 4.56 |
| 6 | F200 |  | Paranoid schizophrenia | | 61 | 4.56 |
| 7 | F195 |  | Mental and behavioural disorders due to multiple drug use and use of other psychoactive substances: Psychotic disorder | | 52 | 3.88 |
| 8 | F319 |  | Bipolar affective disorder, unspecified | | 33 | 2.46 |
| 9 | F603 |  | Emotionally unstable personality disorder | | 31 | 2.32 |
| 10 | F311 |  | Bipolar affective disorder, current episode manic without psychotic symptoms | | 26 | 1.94 |
| ED Visits | | | | | | |
| 1 | F209 |  | Schizophrenia, unspecified | | 281 | 10.77 |
| 2 | F29 |  | Unspecified nonorganic psychosis | | 226 | 8.66 |
| 3 | F155 |  | Mental and behavioural disorders due to use of other stimulants, including caffeine: Psychotic disorder | | 79 | 3.03 |
| 4 | F419 |  | Anxiety disorder, unspecified | | 79 | 3.03 |
| 5 | F259 |  | Schizoaffective disorder, unspecified | | 71 | 2.72 |
| 6 | F430 |  | Acute stress reaction | | 63 | 2.41 |
| 7 | F329 |  | Depressive episode, unspecified | | 62 | 2.38 |
| 8 | F151 |  | Mental and behavioural disorders due to use of other stimulants, including caffeine: Harmful use | | 61 | 2.34 |
| 9 | F195 |  | Mental and behavioural disorders due to multiple drug use and use of other psychoactive substances: Psychotic disorder | | 58 | 2.22 |
| 10 | F603 |  | Emotionally unstable personality disorder | | 55 | 2.11 |
| Claims | | | | | | |
| 1 | 295 |  | schizophrenic disorders* | | 3295 | 19.82 |
| 2 | 296 |  | affective psychoses* | | 1406 | 8.46 |
| 3 | 298.9 |  | psychosis nos | | 1076 | 6.47 |
| 4 | 304 |  | drug dependence* | | 1054 | 6.34 |
| 5 | 311 |  | depressive disorder nec | | 827 | 4.97 |
| 6 | 295.3 |  | paranoid schizophrenia* | | 725 | 4.36 |
| 7 | 304 |  | opioid type dependence* | | 632 | 3.8 |
| 8 | 301.8 |  | other personality dis* | | 558 | 3.36 |
| 9 | 295.7 |  | schizoaffective type* | | 456 | 2.74 |
| 10 | 300 |  | neurotic disorders* | | 374 | 2.25 |
| ATC Dispenses | | | | | | |
| 1 | N05AH04 |  | Antipsychotics | Quetiapine | 2328 | 5.11 |
| 2 | N03AG01 |  | Antiepileptics | Valproic Acid | 2323 | 5.1 |
| 3 | N05AX08 |  | Psycholeptics | Risperidone | 2283 | 5.01 |
| 4 | N05AH03 |  | Antipsychotics | Olanzapine | 2120 | 4.65 |
| 5 | N03AX11 |  | Antiepileptics | Topiramate | 2067 | 4.54 |
| 6 | N05CF01 |  | Psycholeptics | Zopiclone | 1983 | 4.35 |
| 7 | N04AC01 |  | Anti-Parkinson Drugs | Benzatropine | 1798 | 3.95 |
| 8 | N07BC51 |  | Drugs Used in Opioid Dependence | Buprenorphine | 1714 | 3.76 |
| 9 | N03AE01 |  | Antiepileptics | Clonazepam | 1641 | 3.6 |
| 10 | N05AX12 |  | Antipsychotics | Aripiprazole | 1587 | 3.48 |

***Latent Class 2: Actively Managed Middle-aged Patients***

| Rank | Code |  | Description | | Freq. | Percent |
| --- | --- | --- | --- | --- | --- | --- |
|  |  |  |  | |  |  |
| Hospitalizations | | | | | | |
| 1 | F209 |  | Schizophrenia, unspecified | | 84 | 20.14 |
| 2 | F259 |  | Schizoaffective disorder, unspecified | | 33 | 7.91 |
| 3 | F252 |  | Schizoaffective disorder, mixed type | | 19 | 4.56 |
| 4 | F200 |  | Paranoid schizophrenia | | 16 | 3.84 |
| 5 | F29 |  | Unspecified nonorganic psychosis | | 16 | 3.84 |
| 6 | F251 |  | Schizoaffective disorder, depressive type | | 9 | 2.16 |
| 7 | F319 |  | Bipolar affective disorder, unspecified | | 9 | 2.16 |
| 8 | Z508 |  | Care involving use of other rehabilitation procedures | | 9 | 2.16 |
| 9 | F314 |  | Bipolar affective disorder, current episode severe depression without psychotic symptoms | | 7 | 1.68 |
| 10 | F322 |  | Severe depressive episode without psychotic symptoms | | 7 | 1.68 |
| ED Visits | | | | | | |
| 1 | F209 |  | Schizophrenia, unspecified | | 71 | 10.89 |
| 2 | F29 |  | Unspecified nonorganic psychosis | | 35 | 5.37 |
| 3 | F100 |  | Mental and behavioural disorders due to use of alcohol: Acute intoxication | | 16 | 2.45 |
| 4 | F259 |  | Schizoaffective disorder, unspecified | | 15 | 2.3 |
| 5 | F329 |  | Depressive episode, unspecified | | 14 | 2.15 |
| 6 | F419 |  | Anxiety disorder, unspecified | | 13 | 1.99 |
| 7 | F220 |  | Delusional disorder | | 12 | 1.84 |
| 8 | R104 |  | Other and unspecified abdominal pain | | 12 | 1.84 |
| 9 | Z512 |  | Other chemotherapy | | 12 | 1.84 |
| 10 | F430 |  | Acute stress reaction | | 11 | 1.69 |
| Claims | | | | | | |
| 1 | 295 |  | schizophrenic disorders* | | 1692 | 16.34 |
| 2 | 296 |  | affective psychoses* | | 876 | 8.46 |
| 3 | 311 |  | depressive disorder nec | | 472 | 4.56 |
| 4 | 295.3 |  | paranoid schizophrenia* | | 328 | 3.17 |
| 5 | 304 |  | drug dependence* | | 284 | 2.74 |
| 6 | 304 |  | opioid type dependence* | | 264 | 2.55 |
| 7 | 295 |  | simple schizophrenia* | | 260 | 2.51 |
| 8 | 300 |  | neurotic disorders* | | 248 | 2.39 |
| 9 | 299.9 |  | early chld psychosis nos* | | 225 | 2.17 |
| 10 | 295.7 |  | schizoaffective type* | | 224 | 2.16 |
| ATC Dispenses | | | | | | |
| 1 | N05AH04 |  | Antipsychotics | Quetiapine | 6647 | 5.06 |
| 2 | A02BC02 |  | Proton Pump Inhibitors | Pantoprazole | 6550 | 4.99 |
| 3 | N03AE01 |  | Antiepileptics | Clonazepam | 4880 | 3.71 |
| 4 | N05CF01 |  | Psycholeptics | Zopiclone | 4057 | 3.09 |
| 5 | N03AG01 |  | Antiepileptics | Valproic Acid | 3878 | 2.95 |
| 6 | N05AX08 |  | Psycholeptics | Risperidone | 3660 | 2.79 |
| 7 | N03AX12 |  | Other Antiepileptics | Gabapentin | 3300 | 2.51 |
| 8 | N05AH02 |  | Antipsychotics | Clozapine | 2509 | 1.91 |
| 9 | N05AH03 |  | Antipsychotics | Olanzapine | 2441 | 1.86 |
| 10 | A10BA02 |  | Biguanides | Metformin | 2383 | 1.81 |

***Latent Class 3: Elderly Patients with Multiple Chronic Conditions and Polypharmacy***

| Rank | Code |  | Description | | Freq. | Percent |
| --- | --- | --- | --- | --- | --- | --- |
|  |  |  |  | |  |  |
| Hospitalizations | | | | | | |
| 1 | Z508 |  | Care involving use of other rehabilitation procedures | | 43 | 5.02 |
| 2 | F259 |  | Schizoaffective disorder, unspecified | | 38 | 4.43 |
| 3 | J441 |  | Chronic obstructive pulmonary disease with acute exacerbation, unspecified | | 33 | 3.85 |
| 4 | F209 |  | Schizophrenia, unspecified | | 31 | 3.62 |
| 5 | N390 |  | Urinary tract infection, site not specified | | 24 | 2.8 |
| 6 | F252 |  | Schizoaffective disorder, mixed type | | 22 | 2.57 |
| 7 | F319 |  | Bipolar affective disorder, unspecified | | 22 | 2.57 |
| 8 | J440 |  | Chronic obstructive pulmonary disease with acute lower respiratory infection | | 21 | 2.45 |
| 9 | F03 |  | Unspecified dementia | | 19 | 2.22 |
| 10 | J690 |  | Pneumonitis due to food and vomit | | 19 | 2.22 |
| ED Visits | | | | | | |
| 1 | N390 |  | Urinary tract infection, site not specified | | 51 | 3.52 |
| 2 | R074 |  | Chest pain, unspecified | | 48 | 3.31 |
| 3 | Z512 |  | Other chemotherapy | | 48 | 3.31 |
| 4 | R104 |  | Other and unspecified abdominal pain | | 44 | 3.03 |
| 5 | J441 |  | Chronic obstructive pulmonary disease with acute exacerbation, unspecified | | 42 | 2.9 |
| 6 | Z452 |  | Adjustment and management of vascular access device | | 38 | 2.62 |
| 7 | F329 |  | Depressive episode, unspecified | | 32 | 2.21 |
| 8 | R53 |  | Malaise and fatigue | | 31 | 2.14 |
| 9 | F209 |  | Schizophrenia, unspecified | | 29 | 2 |
| 10 | F319 |  | Bipolar affective disorder, unspecified | | 29 | 2 |
| Claims | | | | | | |
| 1 | 295 |  | schizophrenic disorders* | | 1102 | 9.56 |
| 2 | 311 |  | depressive disorder nec | | 973 | 8.44 |
| 3 | 290 |  | senile/presenile psychos* | | 657 | 5.7 |
| 4 | 296 |  | affective psychoses* | | 452 | 3.92 |
| 5 | 780 |  | general symptoms* | | 394 | 3.42 |
| 6 | 296 |  | manic dis, singl episode* | | 340 | 2.95 |
| 7 | 300 |  | neurotic disorders* | | 278 | 2.41 |
| 8 | 250 |  | diabetes mellitus* | | 224 | 1.94 |
| 9 | 295.7 |  | schizoaffective type* | | 205 | 1.78 |
| 10 | 293 |  | transient org mental dis* | | 191 | 1.66 |
| ATC Dispenses | | | | | | |
| **Rank** | **ATC Code** |  | **Drug Class** | **Generic Name** | **Freq** | **Percent** |
| 1 | N05AH04 |  | Antipsychotics | Quetiapine | 7568 | 5.44 |
| 2 | A02BC02 |  | Proton Pump Inhibitors | Pantoprazole (acid reflux) | 6080 | 4.37 |
| 3 | H03AA01 |  | Thyroid Therapy | Levothyroxine sodium | 5098 | 3.66 |
| 4 | N05CF01 |  | Psycholeptics | Zopiclone | 3827 | 2.75 |
| 5 | N03AX12 |  | Other Antiepileptics | Gabapentin | 3632 | 2.61 |
| 6 | N03AG01 |  | Antiepileptics | Valproic Acid | 3411 | 2.45 |
| 7 | B01AC06 |  | Antithrombotic Agents (Platelet aggregation inhibitors excl. heparin) | Acetylsalicyclic Acid | 3197 | 2.3 |
| 8 | A10BA02 |  | Biguanides | Metformin (diabetes) | 3173 | 2.28 |
| 9 | N03AE01 |  | Antiepileptics | Clonazepam | 2857 | 2.05 |
| 10 | N05AX08 |  | Psycholeptics | Risperidone | 2765 | 1.99 |

***Latent Class 4: Unstably Housed Males with Low Treatment Rates***

| Rank | Code |  | Description | | Freq. | Percent |
| --- | --- | --- | --- | --- | --- | --- |
| Hospitalizations | | | | | | |
| 1 | F209 |  | Schizophrenia, unspecified | | 82 | 10.17 |
| 2 | F259 |  | Schizoaffective disorder, unspecified | | 49 | 6.08 |
| 3 | F252 |  | Schizoaffective disorder, mixed type | | 32 | 3.97 |
| 4 | F200 |  | Paranoid schizophrenia | | 24 | 2.98 |
| 5 | F103 |  | Mental and behavioural disorders due to use of alcohol: Withdrawal state | | 21 | 2.61 |
| 6 | F311 |  | Bipolar affective disorder, current episode manic without psychotic symptoms | | 18 | 2.23 |
| 7 | F102 |  | Mental and behavioural disorders due to use of alcohol: Dependence syndrome | | 17 | 2.11 |
| 8 | F29 |  | Unspecified nonorganic psychosis | | 16 | 1.99 |
| 9 | J441 |  | Chronic obstructive pulmonary disease with acute exacerbation, unspecified | | 16 | 1.99 |
| 10 | F319 |  | Bipolar affective disorder, unspecified | | 15 | 1.86 |
| ED Visits | | | | | | |
| 1 | F100 |  | Mental and behavioural disorders due to use of alcohol: Acute intoxication | | 141 | 6.01 |
| 2 | Z512 |  | Other chemotherapy | | 131 | 5.58 |
| 3 | F209 |  | Schizophrenia, unspecified | | 99 | 4.22 |
| 4 | F101 |  | Mental and behavioural disorders due to use of alcohol: Harmful use | | 60 | 2.56 |
| 5 | F419 |  | Anxiety disorder, unspecified | | 58 | 2.47 |
| 6 | R074 |  | Chest pain, unspecified | | 51 | 2.17 |
| 7 | F329 |  | Depressive episode, unspecified | | 47 | 2 |
| 8 | R104 |  | Other and unspecified abdominal pain | | 47 | 2 |
| 9 | Z760 |  | Issue of repeat prescription | | 46 | 1.96 |
| 10 | F151 |  | Mental and behavioural disorders due to use of other stimulants, including caffeine: Harmful use | | 40 | 1.7 |
| Claims | | | | | | |
| 1 | 295 |  | schizophrenic disorders* | | 1264 | 14.37 |
| 2 | 296 |  | affective psychoses* | | 383 | 4.35 |
| 3 | 311 |  | depressive disorder nec | | 380 | 4.32 |
| 4 | 295.3 |  | paranoid schizophrenia* | | 321 | 3.65 |
| 5 | 304 |  | drug dependence* | | 306 | 3.48 |
| 6 | 780 |  | general symptoms* | | 294 | 3.34 |
| 7 | 295.7 |  | schizoaffective type* | | 288 | 3.27 |
| 8 | 300 |  | neurotic disorders* | | 226 | 2.57 |
| 9 | 304 |  | opioid type dependence* | | 207 | 2.35 |
| 10 | 585 |  | chronic renal failure# | | 165 | 1.88 |
| ATC Dispenses | | | | | | |
| 1 | A02BC02 |  | Proton Pump Inhibitors | Pantoprazole | 5168 | 4.92 |
| 2 | N05AH04 |  | Antipsychotics | Quetiapine | 4807 | 4.57 |
| 3 | N05AH03 |  | Antipsychotics | Olanzapine | 4351 | 4.14 |
| 4 | N05CF01 |  | Psycholeptics | Zopiclone | 3649 | 3.47 |
| 5 | N03AX12 |  | Other Antiepileptics | Gabapentin | 3490 | 3.32 |
| 6 | N03AE01 |  | Antiepileptics | Clonazepam | 3281 | 3.12 |
| 7 | N03AG01 |  | Antiepileptics | Valproic Acid | 2566 | 2.44 |
| 8 | B01AC06 |  | Antithrombotic Agents (Platelet aggregation inhibitors excl. heparin) | Acetylsalicyclic Acid | 2470 | 2.35 |
| 9 | N04AC01 |  | Anti-Parkinson Drugs | Benzatropine | 1956 | 1.86 |
| 10 | C10AA05 |  | Lipid Modifying Agents, Plain | Atorvastatin | 1848 | 1.76 |

***Latent Class 5: Unstably Housed Females with High Acute Care Use and Low Treatment Rates***

| Rank | Code |  | Description | | Freq. | Percent |
| --- | --- | --- | --- | --- | --- | --- |
| Hospitalizations | | | | | | |
| 1 | F209 |  | Schizophrenia, unspecified | | 50 | 7.17 |
| 2 | F259 |  | Schizoaffective disorder, unspecified | | 48 | 6.89 |
| 3 | F603 |  | Emotionally unstable personality disorder | | 41 | 5.88 |
| 4 | F29 |  | Unspecified nonorganic psychosis | | 33 | 4.73 |
| 5 | F432 |  | Adjustment disorders | | 24 | 3.44 |
| 6 | F319 |  | Bipolar affective disorder, unspecified | | 22 | 3.16 |
| 7 | F252 |  | Schizoaffective disorder, mixed type | | 21 | 3.01 |
| 8 | F322 |  | Severe depressive episode without psychotic symptoms | | 21 | 3.01 |
| 9 | F329 |  | Depressive episode, unspecified | | 17 | 2.44 |
| 10 | F311 |  | Bipolar affective disorder, current episode manic without psychotic symptoms | | 16 | 2.3 |
| ED Visits | | | | | | |
| 1 | Z512 |  | Other chemotherapy | | 146 | 8.19 |
| 2 | F329 |  | Depressive episode, unspecified | | 77 | 4.32 |
| 3 | R104 |  | Other and unspecified abdominal pain | | 63 | 3.54 |
| 4 | F603 |  | Emotionally unstable personality disorder | | 58 | 3.25 |
| 5 | F29 |  | Unspecified nonorganic psychosis | | 52 | 2.92 |
| 6 | F209 |  | Schizophrenia, unspecified | | 48 | 2.69 |
| 7 | F419 |  | Anxiety disorder, unspecified | | 32 | 1.8 |
| 8 | F430 |  | Acute stress reaction | | 28 | 1.57 |
| 9 | F432 |  | Adjustment disorders | | 28 | 1.57 |
| 10 | F259 |  | Schizoaffective disorder, unspecified | | 27 | 1.52 |
| Claims | | | | | | |
| 1 | 296 |  | affective psychoses* | | 637 | 10.97 |
| 2 | 295 |  | schizophrenic disorders* | | 502 | 8.65 |
| 3 | 311 |  | depressive disorder nec | | 348 | 5.99 |
| 4 | 304 |  | drug dependence* | | 277 | 4.77 |
| 5 | 780 |  | general symptoms* | | 188 | 3.24 |
| 6 | 300 |  | anxiety states* | | 156 | 2.69 |
| 7 | 301.8 |  | other personality dis* | | 144 | 2.48 |
| 8 | 300 |  | neurotic disorders* | | 118 | 2.03 |
| 9 | 786 |  | resp sys/oth chest symp* | | 99 | 1.71 |
| 10 | 295.7 |  | schizoaffective type* | | 91 | 1.57 |
| ATC Dispenses | | | | | | |
| 1 | N05AH04 |  | Antipsychotics | Quetiapine | 4890 | 5.45 |
| 2 | A02BC02 |  | Proton Pump Inhibitors | Pantoprazole | 4011 | 4.47 |
| 3 | N03AX12 |  | Other Antiepileptics | Gabapentin | 3948 | 4.4 |
| 4 | N06AX05 |  | Antidepressants | Trazodone | 3021 | 3.36 |
| 5 | N03AE01 |  | Antiepileptics | Clonazepam | 2681 | 2.99 |
| 6 | N05CF01 |  | Psycholeptics | Zopiclone | 2444 | 2.72 |
| 7 | H03AA01 |  | Thyroid Therapy | Levothyroxine sodium | 2252 | 2.51 |
| 8 | N05AX08 |  | Psycholeptics | Risperidone | 2237 | 2.49 |
| 9 | N06AB06 |  | Antidepressants | Sertraline | 1934 | 2.15 |
| 10 | N03AX11 |  | Antiepileptics | Topiramate | 1928 | 2.15 |
